# Hematological manifestations in systemic lupus erythematosus: a retrospective cross-sectional study from a tertiary care center in Nepal

**DOI:** 10.1097/MS9.0000000000003765

**Published:** 2025-08-29

**Authors:** Krishna Adhikari, Himal Karki, Pradeep Shrestha, Keshav Raj Sigdel, Lucky Sharma, Buddhi Prasad Paudel

**Affiliations:** Department of Internal Medicine, Patan Academy of Health Sciences, Lalitpur, Nepal

**Keywords:** hematological manifestations, Nepal, organ involvement, systemic lupus erythematosus

## Abstract

**Background:**

Hematological abnormalities are common in systemic lupus erythematosus and anemia is the most common hematological manifestation. Studies investigating hematological disorders in Nepali SLE patients are lacking. This study is aimed to understand hematological abnormalities in South Asian population especially Nepali SLE patients.

**Methods:**

A descriptive cross-sectional study was conducted at a tertiary care center in Nepal after obtaining ethical approval from 2019 January to 2023 December. All the patients diagnosed based upon 2019 European League of Associations for Rheumatology and American College of Rheumatology (2019 EULAR/ACR) criteria were included in study excluding those with underlying blood disorders. Hospital records and electronic health records were reviewed retrospectively. A structured proforma was used to collect the data and Microsoft Excel was used for entering the data. Statistical Package for Social Sciences (SPSS) version 16.0 was used for descriptive analysis.

**Results:**

The mean age of 99 SLE patients was 29.31 ± 11.01 years (95% CI, 27.12–31.51). Females were 87 (87.88%) with 38 (38.38%) were of age group 21–30 years. Hematological abnormalities were present in 92 (92.92%) patients; anemia was present in 88 (88.89%) patients followed by lymphopenia, leukopenia, and thrombocytopenia in 56 (56.57%), 24 (24.24%), and 14 (14.14%) patients, respectively. Arthritis was present in 67 (67.68%) and mucocutaneous manifestations in 63 (63.63%) patients. Fifty-nine (59.60%) patients had renal involvement. Arthritis and renal involvement were common across all hematological abnormalities.

**Conclusion:**

Hematological abnormalities especially anemia is much more common than previously reported. Clinician should assess the hematological disorders in SLE patients. Prospective multicenter studies are required to relate organ involvement and hematological manifestations.

## Introduction

Hematological abnormalities are common in systemic lupus erythematosus (SLE) with anemia being the most common manifestation^[1]^. Studies among Indian SLE patients reported hematological abnormalities ranging 70–83%, with anemia up to 87%.^[2–4]^ A study done in Nepal by Paudyal et.al showed that hematological abnormalities were present in 44% of the patients[5]. A recent review on SLE reported that anemia was present in 44–100% of patients with SLE in Nepal[6]. As hematological abnormalities may occur alone or in combination at the onset of the disease, careful interpretation of the hematological abnormalities are important for diagnosing the disease, assessing the disease activity and treatment response as well as ruling out the other hematological diseases[7].

**HIGHLIGHTS**

- Anemia is most common hematological manifestations among SLE patients.
- Arthritis and mucocutaneous manifestations are frequent clinical presentations in SLE.
- Hematological abnormalities in SLE are often associated with arthritis and renal involvement.

However, studies investigating the hematological abnormalities in South Asian population especially in Nepali SLE patients are sparse and have not looked into their relation to organ involvement[5]. Therefore, this study aims to provide the basic knowledge and understanding of hematological abnormalities and organ involvement in SLE in patients presenting to a tertiary care center. This retrospective study has been reported in line with the STROCSS guidelines[8].

## Methods

A descriptive cross-sectional study was conducted after obtaining ethical approval in a tertiary care center in Nepal among SLE patients diagnosed as per 2019 European League of Associations for Rheumatology and American College of Rheumatology (2019 EULAR/ACR) criteria from 2019 January to 2023 December[9]. The electronic hospital recording system and patients’ hospital files were reviewed to collect data retrospectively. Data on age, sex and laboratory values of hemoglobin (Hb), white blood cell count (WBC), differential count, platelet count, and erythrocyte sedimentation rate (ESR) as well as clinical variables, including the presence of malar rash, discoid rash, alopecia, oral ulcers, pericarditis, respiratory involvement, gastrointestinal involvement, kidney involvement, arthritis, and neurological involvement were collected using proforma. Patients with underlying hematological disorders such as hemophilia, leukemia, or lymphoma, those with conditions that could independently alter hematological parameters, and those with incomplete records were excluded.

Anemia was defined as a Hb of less than 13 g/dL in males and less than 12 g/dL in females, while leukopenia was defined as a WBC of less than 4000 per cubic millimeter (cu.mm)^[1]^. Thrombocytopenia was defined as a platelet count of less than 100,000 per cubic millimeter. Similarly, lymphopenia and neutropenia were defined as absolute lymphocyte count less than 1500 per cu.mm and absolute neutrophil count less than 1800 per cu.mm[1]. Arthritis, hematological involvement, renal involvement, gastrointestinal involvement, pulmonary involvement, pericarditis and neurological involvement were diagnosed based upon the 2019 EULAR/ACR criteria[9]. Data were entered into Microsoft Excel 2013 and imported into Statistical Package for Social Sciences (SPSS) version 16.0. Laboratory values were categorized into anemia, leukopenia and thrombocytopenia as mentioned. Descriptive statistics was used to summarize the results from the data.

Ethical approval for this study (reference number: drs2412171972) was provided by the Institutional Review Committee of Patan Academy of Health Sciences (IRC-PAHS). The study was registered in ClinicalTrials.gov with registration number: NCT06936007. This study has been reported as per STROCSS 2025 guideline[8].

## Results

A total of 99 patients with a diagnosis of SLE were included in the study. The mean age (±SD) of the patient was 29.31 ± 11.01 years (27.12–31.51 years at 95% CI). Females were 87 (87.88%) and males were 12 (12.12%). Most of the patients were in age group 21–30 years with 38 (38.38%) (Table 1). The mean hemoglobin of the SLE patients was 9.13 ± 2.21 g/dl (8.69–9.57 at 95% CI). Similarly, mean values of WBC, platelets, neutrophil, and lymphocyte was 7.08 ± 4.14 × 10^3^ per cu.mm, 194.34 ± 102.31 × 10^3^ per cu.mm, 5.33 ± 3.68 × 10^3^ per cu.mm, and 1.59 ± 0.92 × 10^3^ per cu.mm, respectively.

**Table 1:** Demographics characteristics of systemic lupus erythematosus patients (n = 99)

Characteristics	Frequency	Percentage
Sex		
Male	12	12.12%
Female	87	87.88%
Age group		
≤20 years	21	21.21%
21–30 years	38	38.38%
31–40 years	26	26.26%
41–50 years	9	9.09%
51–60 years	3	3.03%
>60 years	2	2.02%

Among 99 patients, hematological manifestations were found in 92 (92.92%) patients. Anemia was the most frequently observed, present in 88 (88.89%) patients, followed by lymphopenia and leukopenia in 56 (56.57%) and 24 (24.24%) patients, respectively (Table 2). Thrombocytopenia and pancytopenia were present in 14 (14.14%) patients each.

**Table 2:** Hematological abnormalities in patients with systemic lupus erythematosus (n = 99)

Parameter	Frequency	Percentage
Anemia	88	88.89%
Lymphopenia	56	56.57%
Leukopenia	24	24.24%
Neutropenia	15	15.15%
Thrombocytopenia	14	14.14%
Pancytopenia	14	14.14%

Sixty-seven (67.68%) patients with SLE had arthritis, followed by 63 (63.64%) patients who had mucocutaneous manifestation in the form of malar rash, discoid rash, alopecia, or oral ulcer. Kidney was involved in 59 (59.60%) patients with SLE. Oral ulcers and alopecia were frequent mucocutaneous manifestations of the disease (Table 3). Neurological involvement was least reported, present only in 8 (8.70%) patients.

**Table 3:** Frequency of organ involvement in patients with systemic lupus erythematosus (n = 99)

Organ involvement	Frequency	Percentage
Hematological	92	92.93%
Malar rash	26	26.26%
Discoid rash	21	21.21%
Alopecia	31	31.31%
Oral ulcer	37	37.37%
Arthritis	67	67.68%
Gastrointestinal	19	19.19%
Pulmonary	11	11.11%
Pericarditis	10	10.10%
Renal involvement	59	59.60%
Neurological involvement	8	8.08%

Arthritis and renal involvement were most frequent organ involvement found across all the hematological abnormalities (Table 4). Oral ulcers and alopecia were also common in patients with anemia and pancytopenia. Neurological involvement was least frequent in these patients.

**Table 4:** Hematological abnormalities and organ involvement in SLE patients

Hematological abnormalities	Organ involvement	Frequency	Percentage
Anemia	Arthritis	61	69.32%
	Renal involvement	54	61.36%
	Oral ulcers	34	38.64%
Leukopenia	Arthritis	15	62.50%
	Renal involvement	14	58.33%
	Oral ulcers	10	41.67%
Thrombocytopenia	Arthritis	11	78.57%
	Discoid rash	7	50%
	aOral ulcers/renal involvement/gastrointestinal	5	35.71%
Pancytopenia	bRenal involvement/arthritis	10	71.43%
	Oral ulcers	6	42.86%
	Discoid rash	5	35.71%
Overall hematological abnormalities	Arthritis	63	68.48%
	Renal involvement	56	60.87%
	Oral ulcers	35	38.04%

a Indicates oral ulcers, renal involvement and gastrointestinal involvement was present in 5 patients each

b indicates renal involvement and arthritis was present in 10 patients each

## Discussion

This study showed hematological abnormalities are common in SLE patients. Ninety-two percent of patient in our study had at least one hematological disorder, anemia being the most common manifestation (which was found in 88.89% of SLE patients). The mean hemoglobin level in SLE patient in this study was 9.13 ± 2.21 g/dl which is lower than normal value. The finding is similar to studies from low- and middle-income countries (LMIC) which reported prevalence of hematological abnormalities ranging from 83% to 93% and anemia ranging from 56% to 98% in SLE patients.^[2,10–12]^ However, prevalence of hematological abnormalities and anemia in our study is higher than in a previous study conducted by Paudyal *et al* in Nepali SLE patients which reported hematological abnormalities in 44% of the patients and anemia was present in 28%[5]. Also, a study from a higher-income countries (HIC) reported anemia in only 52% of SLE patients[13]. Anemia in SLE is multifactorial and common mechanisms include the immune-mediated destruction of red blood cells as well as nutritional deficiencies such as iron, vitamin B12 or folic acid deficiency[14]. The high prevalence anemia in our study might be the nutritional deficiencies which are prevalent in LMIC[15]. Nutritional assessment and targeted supplementation in SLE patient along with efforts to strengthen existing nutritional programs would be valuable strategies to reduce anemia-related morbidity, especially in resource-limited settings where such deficiencies are common.

In our study, lymphopenia was observed in twice as many patients as leukopenia. This may be due to the immune-mediated destruction of lymphocytes more than other white blood cells in SLE patients[16]. A study done by Bathina *et al* in Indian SLE patients found leukopenia in 92% of the patients which is much higher than 24.24% reported by our study[10]. Studies had reported mixed result for lymphopenia in SLE patients as well. A study conducted by Sufian *et al* in Bangladeshi SLE patients reported the presence of lymphopenia in much lower number of patients (17.9% versus 56.57%) whereas another study in Indian population revealed lymphopenia in much higher number of patients (85% versus 56.57%)^[10,11]^. The variation in the findings is likely due to immunosuppressive treatment exposure and variation in the disease severity of the patient included in the study. In fact, lymphopenia is considered as hallmark of active and severe SLE disease condition[17].

Thrombocytopenia was observed in 14.14% of patients consistent with the findings from a study done in Turkey and Bangladesh that documented presence in 17.2% and 17.9% of the SLE patients, respectively^[11,18]^. However, it was less than 38% reported by Bathina *et al* in Indian SLE patients[10]. Conversely, a study among South Korean SLE patients by Koh *et al* documented thrombocytopenia in 46.5%[19]. Similar mechanism of immune-mediated destruction as in anemia and leukopenia as well as peripheral destruction of platelets along with decreased production leads to the decreased platelet count[20]. A study done by Galil et.al. concluded that thrombocytopenia is significantly associated with disease activity and has prognostic value to identify aggressive course of disease[21].

Pancytopenia characterized by reduction in Hb, WBC and platelets count was found in 14.14% of SLE patients in our study which was higher than 5.6% reported in Bangladesh[11]. The dysregulated immune system and modified cytokine pattern in SLE causes destruction of the hematopoietic cells in bone marrow leading to hypocellularity and bone marrow suppression causes decreased production of RBC, WBC and platelets[16]. The prevalence of pancytopenia in our study was similar to the studies conducted in India which reported prevalence ranging from 14% to 22%^[2,3]^. There are also documented cases of SLE patients presenting with pancytopenia as initial clinical manifestation^[22,23]^. In addition to that treatment itself can cause pancytopenia in SLE patients and close monitoring is essential.

Different studies have reported variability in the clinical manifestations of the SLE depending upon the study population. A study from Saudi Arabia showed mucocutaneous and musculoskeletal manifestations were common presenting features in 76% and 57% of the SLE patients respectively, whereas hematological manifestations were present in 60%[24]. Similar findings were observed in a study done by Koh *et al* among South Korean SLE patients where 81.6% patients had mucocutaneous lesions, 59.1% had musculoskeletal manifestations and 81.1% had hematological manifestations[19]. In comparison our study reported 67.68% patients had musculoskeletal manifestations that was arthritis followed by 63.64% had mucocutaneous manifestations. However, Kafle *et al* in a review of Nepali SLE patients found mucocutaneous manifestations to be the most common presentation followed by musculoskeletal manifestations[6]. In our study, renal involvement was present in 59.60% of the patients higher than previously documented 44% by Paudyal *et al* in Nepali SLE patients[5]. Conversely, renal involvement among Indian SLE patients range from 58% to 69%^[4,25]^.Previous studies have associated the hematological abnormalities with the organ involvement in SLE patients^[1,26]^. In our study, arthritis, renal involvement and oral ulcers were common among all hematological abnormalities with oral ulcers being common patients with anemia.

Although this study provides some insights into the organ involvement and hematological abnormalities in SLE patients adding to the literature, this study has several limitations. The retrospective design of the study and reliance on hospital records as well as changes in diagnostic and coding practices for identification of SLE may have introduced potential selection bias. Furthermore, nutritional deficiencies such as iron, folate or vitamin B12 deficiency were not assessed, even though they are likely contributors to the high prevalence of anemia observed in settings. Lastly, the single-center design and small sample size limit the generalizability of the study findings to broader populations. Future multicenter studies with prospective design and large sample size are needed to enhance generalizability and validity of the study as well as to provide comprehensive understanding of the hematological manifestations in Nepali SLE patients and its association with organ involvement. Assessment of nutritional status through objective measurement of iron, folate and vitamin B12 levels would enhance the understanding of anemia in SLE patients and use of standardized criteria would be helpful to reduce the biases.

## Conclusion

Hematological manifestations among SLE patients are more common than previously reported by similar studies in Nepal. Anemia is most common hematological manifestation and arthritis is most common clinical presentation. Oral ulcers and alopecia are common mucocutaneous manifestation of SLE frequently present with anemia and pancytopenia. Hematological manifestations especially in young female should prompt for evaluation of SLE. Future studies multicenter, prospective studies with large sample size are required to explore the association between organ involvement and hematological manifestations in Nepali SLE patients.

## Data Availability

The data supporting the findings of this study are available upon request from Editor-in-Chief.

## Research registration unique identifying number (UIN)

ClinicalTrials.gov ID: NCT06936007 (https://clinicaltrials.gov/study/NCT06936007).
